# The combination of ^13^N-ammonia and ^11^C-methionine in differentiation of residual/recurrent pituitary adenoma from the pituitary gland remnant after trans-sphenoidal Adenomectomy

**DOI:** 10.1186/s12885-021-08574-1

**Published:** 2021-07-20

**Authors:** Fangling Zhang, Qiao He, Ganhua Luo, Yali Long, Ruocheng Li, Lei Ding, Xiangsong Zhang

**Affiliations:** 1 Department of Radiology, Hospital of Stomatology, Guanghua School of Stomatology, Sun Yat-sen University & Guangdong Provincial Key Laboratory of Stomatology, 56#, Cemetery west Road, Guangzhou, Guangdong Province 510055 People’s Republic of China; 2 Department of Medical Imaging, The First Affiliated Hospital, Sun Yat-sen University, 58#, Zhongshan Er Road, Guangzhou, Guangdong Province 510080 People’s Republic of China

**Keywords:** Residual/recurrent pituitary adenoma, Pituitary gland remnant, ^13^N-ammonia, ^11^C-methionine, PET/CT

## Abstract

**Background:**

This study aimed to assess the clinical usefulness of ^13^N-ammonia and ^11^C- Methionine (MET) positron emission tomography (PET)/ computed tomography (CT) in the differentiation of residual/recurrent pituitary adenoma (RPA) from the pituitary gland remnant (PGR) after trans-sphenoidal adenomectomy.

**Methods:**

Between June 2012 and December 2019, a total of 19 patients with a history of trans-sphenoidal adenomectomy before PET/CT scans and histological confirmation of RPA after additional surgery in our hospital were enrolled in this study. Images were interpreted by visual evaluation and semi-quantitative analysis. In semi-quantitative analysis, the maximum standard uptake value (SUVmax) of the target and gray matter was measured and the target uptake/gray matter uptake (T/G) ratio was calculated.

**Results:**

The T/G ratios of ^13^N-ammonia were significantly higher in PGR than RPA (1.58 ± 0.69 vs 0.63 ± 1.37, *P* < 0.001), whereas the T/G ratios of ^11^C-MET were obviously lower in PGR than RPA (0.78 ± 0.35 vs 2.17 ± 0.54, *P* < 0.001). Using the canonical discriminant analysis, we calculated the predicted accuracy of RPA (100%), PGR (92.9%), and the overall predicted accuracy (96.43%).

**Conclusions:**

The combination of ^13^N-ammonia and ^11^C-MET PET/CT is valuable in the differentiation of RPA from PGR after trans-sphenoidal adenomectomy.

## Background

The main treatment for primary pituitary adenomas is trans-sphenoidal surgery and residual/recurrent pituitary adenoma (RPA) can be seen in many patients [1–3]. Accurate localization of RPA and its differentiation from pituitary gland remnant (PGR) can promote targeted therapy, increase the remission rate and maximize the preservation of pituitary function. Postoperative magnetic resonance imaging (MRI), especially early reexamination, is difficult to interpret due to fluid, hemorrhage, and implanted material [4–6]. Methionine (MET), a substrate for ^11^C labeling to trace increased protein synthesis, is a promising positron emission tomography (PET) tracer for the diagnosis of pituitary adenomas [7–9]. According to previous studies, ^11^C-MET was superior to somatostatin receptor (SSTR) ligand tracer and ^18^F-fluorodeoxyglucose (FDG) since pituitary adenomas are characterized by high amino acid metabolism [10–12]. Our research team previously reported that ^13^N-ammonia was useful in identifying pituitary tissue [13, 14]. The main uptake mechanism is as follows: (1) metabolic trapping of ^13^N-ammonia in pituitary tissue is related to the glutamine synthetase pathway (2) ^13^N-ammonia is a good indicator of pituitary tissue blood flow. The purpose of this study was to retrospectively assess the usefulness of ^13^N-ammonia and ^11^C-MET PET/ computed tomography (CT) in the differentiation of RPA from PGR after trans-sphenoidal adenomectomy.

## Methods

### Patients

We retrospectively reviewed the data of suspected RPA (biological suggestion of active residual/recurrent tumor or MRI demonstration of nonfunctional pituitary adenoma) patients who underwent ^13^N-ammonia and ^11^C-MET PET/CT scans in our center between June 2012 and December 2019. Patients who met the following requirements were included in this study:(1) a clinical history of trans-sphenoidal adenomectomy with histological confirmation before the PET/CT scan; (2) additional surgery after PET/CT imaging and pathological confirmation of RPA after the additional surgery; (3) the position of remaining pituitary tissue was confirmed during surgery or regular imaging and clinical follow-up for at least 1 year. Patients were excluded if they previously received radiation therapy, underwent surgical resection elsewhere or lost follow-up. Eventually a total of 19 patients (14 females and 5 males, mean age: 44.86 ± 15.58 years, range: 18–79 years) were enrolled in this study and 14 of them were confirmed with PGR. Three patients were nonfunctional and 16 patients were hormone-secreting [9 patients produced adrenocorticotropic hormone (ACTH), 5 patients produced prolactin (PRL), 1 patient produced growth hormone (GH), and 1 patient produced follicle-stimulating hormone (FSH)]. This study was approved by the hospital ethics committee and the need for signed informed consent was waived.

### Radiotracer synthesis and PET imaging protocol

^13^N-ammonia and ^11^C-MET were produced in our department by commercially available systems for isotope generation (Ion Beam Applications, Cyclone-10, Belgium) with standard methods. The radiochemical purity was>99%. PET/CT scans were performed with a Gemini GXL-16 scanner (Philips, Netherlands). Five minutes after intravenous injection of 7.4 MBq (0.20 mCi)/kg ^13^N-ammonia or ^11^C-MET, a 10-min serial PET/CT scan using a brain imaging protocol (matrix: 128 × 128 pixels; slice thickness: 1.5 mm; FOV: 180 mm) was initiated. Images were attenuation-corrected with low-dose CT and reconstructed with the Line of Response RAMLA algorithm. ^13^N-ammonia and ^11^C-MET PET/CT were performed with an interval of at least 24 h.

### Imaging analysis

PET images were interpreted by two experienced nuclear physicians independently and reached a consensus.

#### Visual analysis

The uptake of the targets was classified into 3 degrees visually compared to the surrounding normal brain tissue: low uptake, moderate uptake, and high uptake. Moderate and high uptake on ^11^C-MET PET/CT images was considered positive.

#### Semi-quantitative analysis

For each patient, a region of interest (ROI) over the entire target was drawn on the transaxial plane referred to MRI or computerized tomography (CT) images, and the maximum standardized uptake value (SUVmax) was measured. Then another referenced ROI (approximately 10 mm in diameter) was drawn on the normal gray matter of the left prefrontal cortex, gaining the target uptake/gray matter uptake (T/G) ratio.

### Statistical analysis

Statistical analysis was performed using SPSS 20.0 software (IBM SPSS statistics 20.0, Armonk, NY, USA). Measurement data were expressed in form of mean ± standard deviation (SD). First, the T/G ratios of RPA and PGR were compared using Student’s *t*-test for each tracer. Second, the T/G ratios of the two tracers were adopted as multiple variables in the canonical discrimination analysis. By obtaining the canonical discriminant function, each patient could be successfully classified into one group according to the functional result. Finally, cross validation was performed. *P* < 0.05 suggested that difference had statistical significance.

## Results

PGR was identified in 14 patients with PET and could not be identified by any other imaging modality in 5 patients. Of the 14 patients, 8 were determined to have normal function, and 6 were determined to have hypopituitarism. The diameter of RPA ranged from 1.0 to 4.2 cm (1.83 ± 0.73 cm).

### Result of visual analysis

For the 19 patients with RPA, 14 (73.68%), 3(15.79%), 2(10.53%) patients were graded as low, moderate, and high uptake respectively on ^13^N-ammonia PET images and 1 (5.26%), 18 (94.74%) patients were graded as low and high uptake respectively on ^11^C-MET PET images.

For the 14 patients with PGR, 2 (14.29%), 12 (85.71%) patients were graded as moderate and high uptake respectively on ^13^N-ammonia PET images and 8 (57.14%), 5 (35.71%), 1 (7.14%) patients were graded as low, moderate and high uptake respectively on ^11^C-MET PET images.

Of the 19 patients with RPA, MET was positive in 18 patients (18 true positive) and negative in 1 patient (1 false negative; tumor type: ACTH) compared to histological results.

### Result of semi-quantitative analysis

**Fig. 1:**
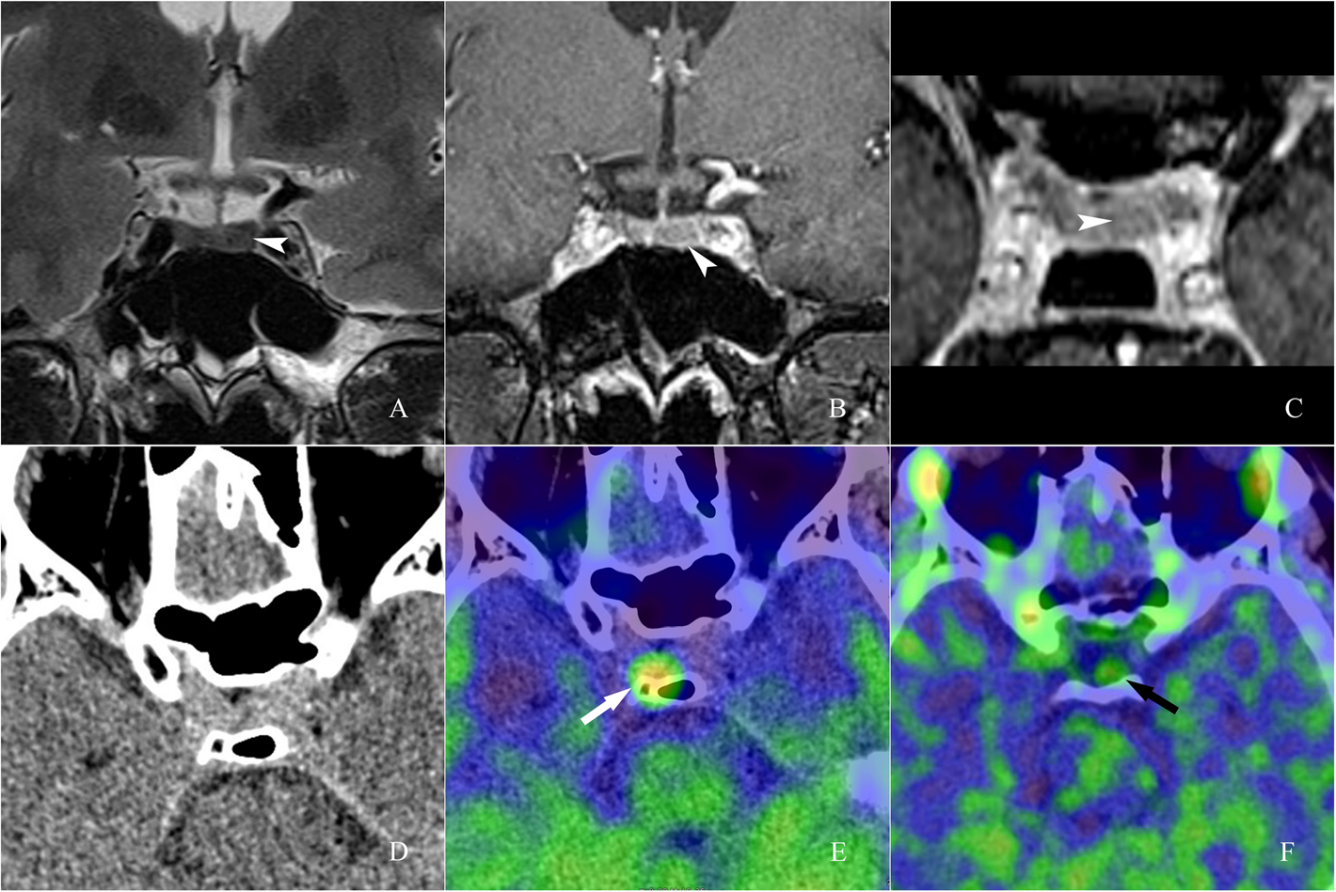
Images of a 20-year-old man with recurrent adrenocorticotropic hormone (ACTH)-secreting pituitary adenoma. A (T2-weighted coronal image), B (T1-weighted contrast-enhanced coronal image) and C (T1-weighted contrast-enhanced axial image) showed a suspicious equisignal lesion in the left part of the sella on plane and enhanced images (arrowhead). A PET/CT scan was recommended by clinician. On ^13^N-ammonia PET/CT, a hypermetabolic region in the right part of the sella was observed, revealing the pituitary gland remnant with normal function (E, white arrow). On ^11^C-MET PET/CT, a moderate-metabolic region in the left part of the sella was observed, revealing the recurrent adenoma (F, black arrow)

**Fig. 2:**
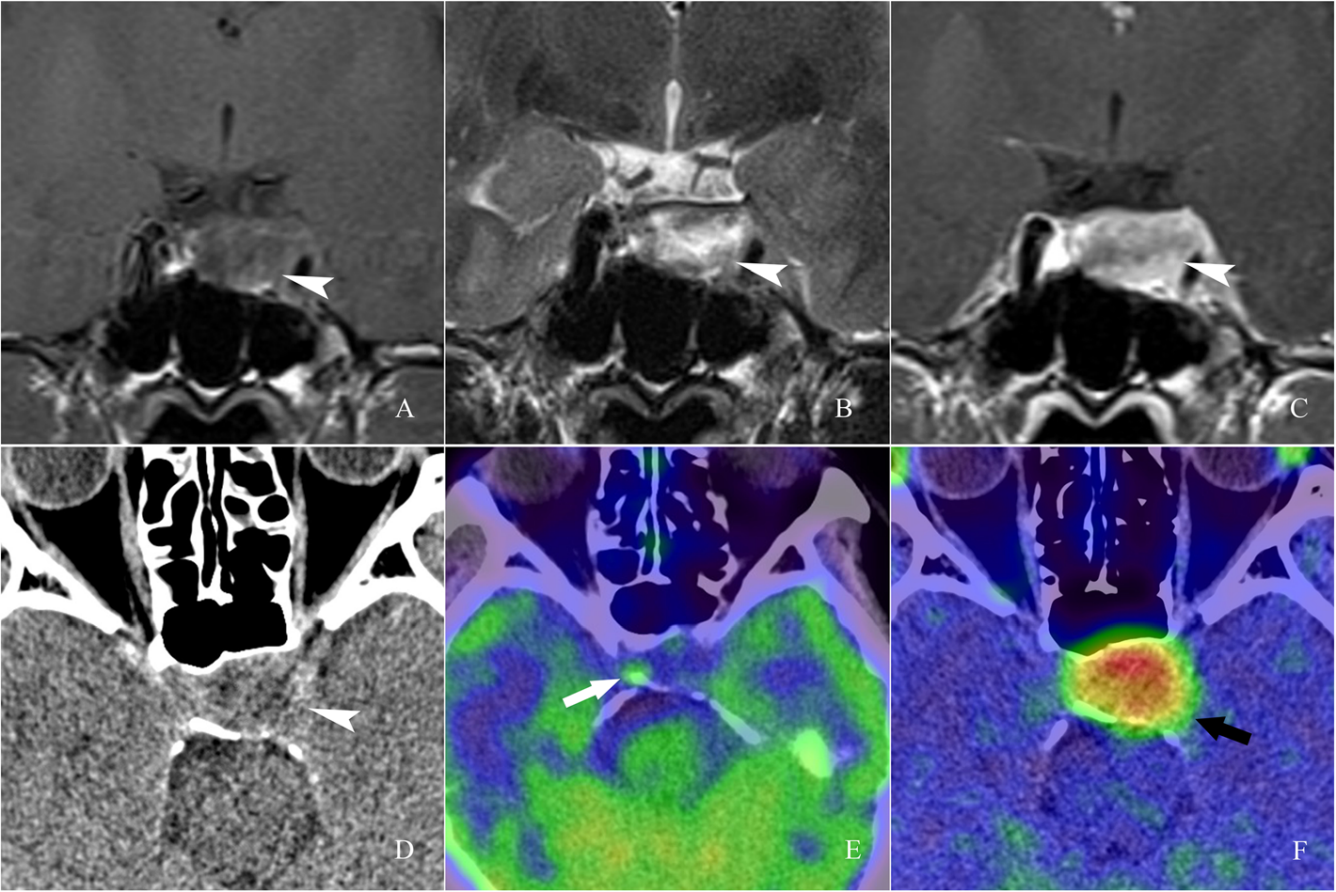
Images of a 37-year-old woman with recurrent prolactin (PRL)-secreting pituitary adenoma. T1-weighted coronal image, T2-weighted coronal image and T1-weighted contrast-enhanced coronal image showed an adenoma in the sella (A, B, C arrowhead). On ^13^N-ammonia PET/CT, a moderate-metabolic region in the right part of the sella was observed, revealing the pituitary gland remnant with hypopituitarism (E, white arrow). On ^11^C-MET PET/CT, an extreme hypermetabolic region in the the sella was observed, which was consistent with the CT and MRI images (F, black arrow)

**Fig. 3:**
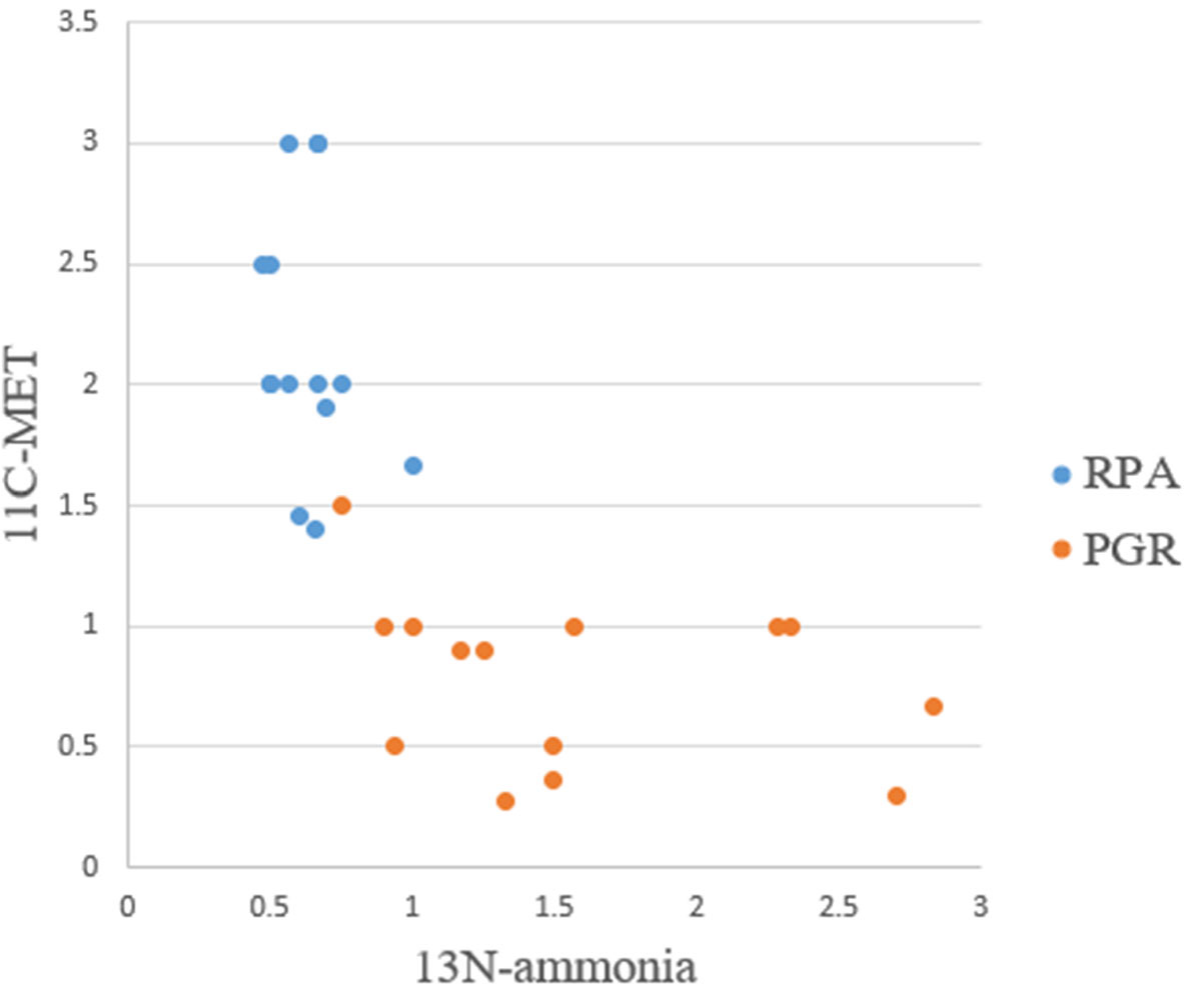
Distribution map of T/G ratios of ^13^N-ammonia and ^11^C-MET. As shown in this figure, a small overlap was observed between RPA (blue spot) and PGR (red spot)

**Fig. 4:**
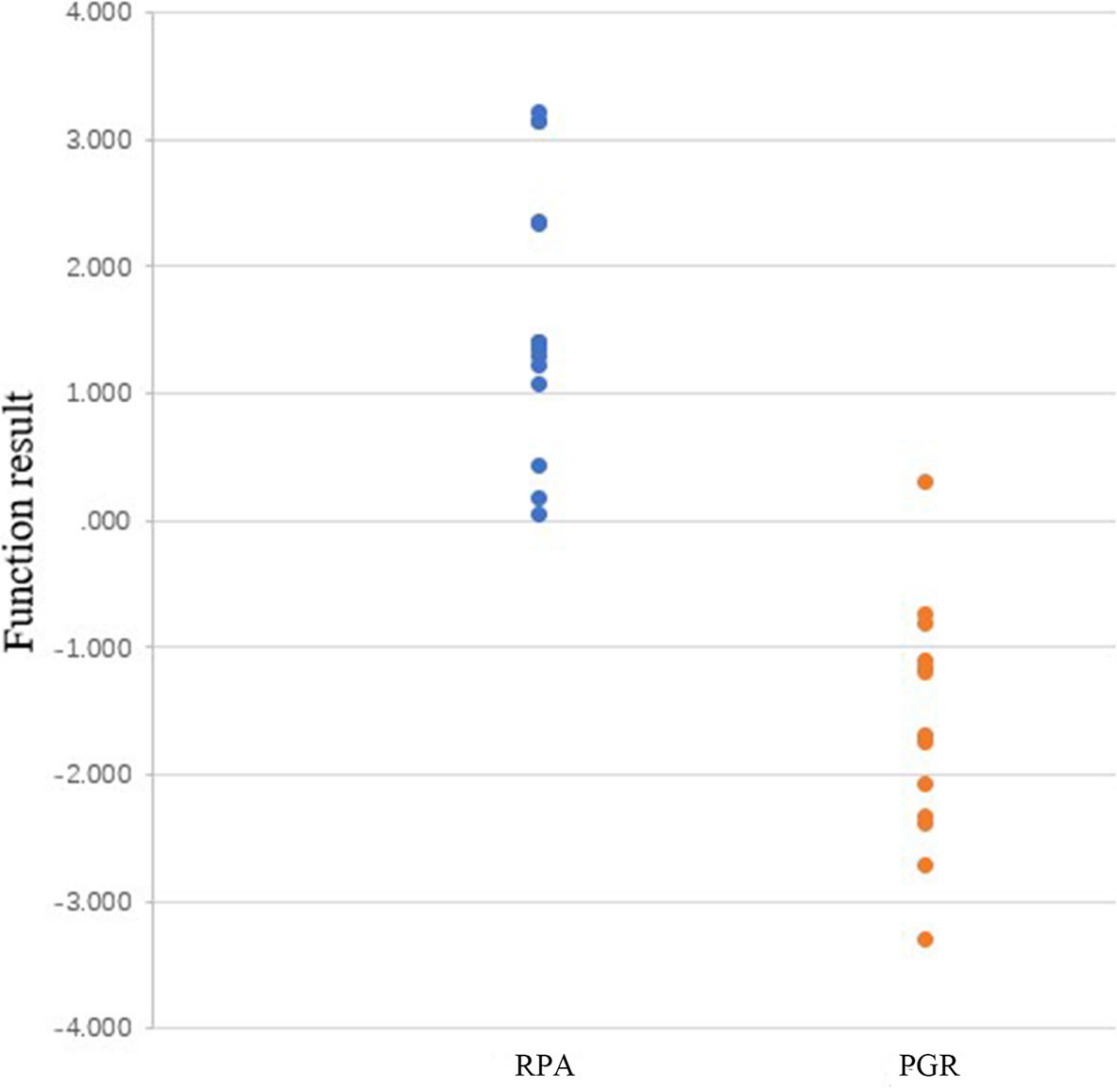
Discriminant function results of the two groups. The functional results of RPA were obviously higher than that of PGR (1.65 ± 1.03 vs − 1.65 ± 0.97). The combination of the two tracers could distinguish these two clinical entities effectively

The T/G ratios of ^13^N-ammonia were significantly higher in PGR than RPA (1.58 ± 0.69 vs 0.63 ± 1.37, *P* < 0.001), however, the T/G ratios of ^11^C-MET were obviously lower in PGR than RPA (0.78 ± 0.35 vs 2.17 ± 0.54, *P* < 0.001) (Figs. 1, 2 and 3). Canonical discriminant analysis with 14 patients whose PRA could be identified showed that the optimal discriminant function was F (x, y) = − 0.814 x + 1.820 y − 1.788, where x represented a T/G ratio of ^13^N-ammonia and y represented a T/G ratio of ^11^C-MET. The functional result of RPA was 1.65 ± 1.03, which was higher than that of PGR (− 1.65 ± 0.97, *P* < 0.001). The predicted accuracy of RPA (100%), PGR (92.9%) and the overall predicted accuracy (96.43%) were calculated. As a result, only 1 PGR was misdiagnosed as RPA (Table 1, Fig. 4).

**Table 1:** Predicted accuracy of the 2 groups by discriminant analysis

		Predicted Group Membership			
		Group	RPA	PGR	Total
**Original**	n (%)	RPA	14 (100)	0 (100)	14 (100)
		PGR	1 (100)	13 (100)	14 (100)
**Cross-validated**	n (%)	RPA	14 (100)	0 (100)	14
		PGR	1 (100)	13 (100)	14 (100)

## Discussion

For patients after trans-sphenoidal pituitary adenomectomy, postoperative follow-up by clinical and imaging methods remains necessary. To our knowledge, MRI is an excellent imaging modality and the top choice in the assessment of pituitary lesions. However, considering the morphological changes of the postoperative area and the confusion about the anatomical structures, MRI is often unable to distinguish RPA from PGR [4–6, 15]. In addition, RPA is particularly more difficult to detect by MRI given its smaller size compared with the primary tumor [6, 16]. PET is a complementary imaging technique for pituitary adenoma evaluation with different tracers [17]. As the most commonly used PET tracer, ^18^F-FDG was described to be unsatisfactory in the diagnosis of pituitary adenomas (especially recurrent cases), probably because FDG uptake is related to tumor malignancy or aggressiveness rather than hormonal production and secretion [18, 19]. Its utility is also limited by low sensitivity and high uptake in surrounding normal brain tissue.

In our study, ^13^N-ammonia demonstrated higher uptake in PGR than RPA and ^11^C-MET had higher uptake in RPA compared with PGR. Thus, ^13^N-ammonia PET played a better role in the identification of PGR, whereas ^11^C-MET PET seems more useful in the detection of RPA.

^13^N-ammonia is not only a good indicator of the blood flow of the pituitary gland, but also a potential tracer to indicate glutamine synthesis metabolism, as reported in our previous studies [20–22]. Pituitary tissue exhibited significantly high uptake of ^13^N-ammonia due to the absence of the blood-brain barrier (BBB), high regional cerebral blood flow (CBF), and high capillary permeability surface area product [22]. Glutamine synthetase (GS) is a catalyst in the glutamine synthesis reaction and its activity was confirmed in the anterior lobe of the pituitarium [23] As for displaying the position of pituitary tissue, the ability of ^13^N-ammonia is comparable to MRI [14]. Even in some cases, ^13^N-ammonia could exhibit potential value when MRI findings were negative. Besides, ^13^N-ammonia is also a promising imaging method to reflect the pituitary function. Hypopituitarism showed decreased ^13^N-ammonia uptake and could be diagnosed in the early stage [13]. In our study, 14 patients were identified with PGR (14/14) on ^13^N-ammonia images.

The usefulness of ^11^C-MET PET/CT in identifying pituitary tumors has been reported in previous studies [10, 11, 24]. Furthermore, BNT Tang and ZZ Feng reported a high positive rate of ^11^C-MET PET/CT in recurrent adenomas regardless of the tumor size and the endocrine subgroups [8, 10], contributing to the efficient management of tumors. RPA was characterized by high amino acid metabolism. The detection rate of ^11^C-MET was very high in this investigation for RPA (18/19), even in nonfunctional adenomas, which was in accordance with previous studies [8, 10, 11]. Only one patient showed low uptake of ^11^C-MET due to extensive cystic degeneration within the tumor. Conversely, PGR showed minimal uptake on ^11^C-MET images. Sex, age, and the menstrual cycle in women can influence the accumulation of ^11^C-MET in the remaining pituitary tissue. Occasionally, the ^11^C-MET uptake in pituitary gland can be confused and result in false positive results. Besides, RPA is not always identified with high uptake because of necrosis, cystic degeneration and hemorrhage. To avoid this phenomenon, another imaging comparison has been suggested to identify residual pituitary tissue [25]. We selected ^13^N-ammonia to locate the pituitary gland based on our previous studies [13, 20–22]. When ^13^N-ammonia was combined with ^11^C-MET, the differential accuracy of these 2 clinical entities was 96.43%. By accurately locating RPA and PGR, ^13^N-ammonia and ^11^C-MET PET can guide surgery and realize the maximal protection of pituitary function. ^68^Ga 1,4,7,10-tetraazacyclododecane-N, N′, N″, N″’-tetraacetic acid-D-Phe1, Tyr3-octreotate (DOTATATE), a novel somatostatin analog, can also be applied in the recognition of remaining pituitary tissue. The accumulation was only lower than that in spleen, kidneys, and adrenal glands [26, 27]. However, limited information has been reported so far. Further studies are needed to explore the efficacy of ^68^Ga-DOTATATE.

One major limitation of the study is that we did not provide the uptake information of scar tissue. Scar tissue may form at the post-operative site, particularly after graft implantation. Although the scar tissue should have no or minimal metabolism as predicted in previous study [5], caution is warranted to interpret it. Then, we were not able to make comparison of conventional MRI and PET in the differentiation of RPA and PGA because not every patient underwent MRI scan in our hospital. Future study should be performed on this problem.

## Conclusions

The combination of ^13^N-ammonia and ^11^C-MET PET/CT is valuable in the differentiation of RPA from PGR after trans-sphenoidal adenomectomy.

## Data Availability

The dataset supporting the conclusions of this article is included within the article. Data and materials during the current study are available from the corresponding author upon reasonable request.

## Abbreviations

RPA: Residual/recurrent pituitary adenoma
PGR: Pituitary gland remnant
MRI: Magnetic resonance imaging
MET: Methionine
SSTR: Somatostatin receptor
^18^F-FDG: ^18^F-fluorodeoxyglucose
GH: Growth hormone
ACTH: Produced adrenocorticotropic hormone
FSH: Follicle-stimulating hormone
ROI: Region of interest
CT: Computerized tomography
SUVmax: Maximum standardized uptake value
BBB: Blood brain barrier
CBF: Cerebral blood flow
GS: Glutamine synthetase
DOTATATE: Etraazacyclododecane-N, N′, N″, N″’-tetraacetic acid-D-Phe1, Tyr3-octreotate
